# Committees and the Shortage of Resident Officers

**Published:** 1914-09-12

**Authors:** 


### COMMITTEES AND THE SHORTAGE OF RESIDENT OFFICERS.

For some years past there has been an undoubted shortage of resident medical officers for the numerous and what at one time were and should still be the much sought after resident posts in our voluntary hospitals. This shortage, it was suggested, was a temporary one due to a falling off in the entries to the medical schools, which it was hoped would be righted with the keen demand for doctors consequent upon the creation of numerous well-paid official posts and on the greater demand for practitioners on the passing of the National Insurance Act, which has been an undoubted financial benefit to many "panel" medical men. The condition of things a year ago was specially dealt with in an article, based on extensive investigation, which appeared in the Educational Number of The Hospital, which was published on September 6, 1913. That article, entitled "The Scarcity of Resident Medical Officers: the Facts, the Cause, and the Remedy," showed that to raise salaries was not sufficient, and also pointed out that the best-administered hospitals had the least difficulty. But to-day, of course, under the present circumstances, the situation in regard to resident medical officers has grown still more complicated.

With the outbreak of the present great war and the sudden demand for medical men, the problem has been reopened in its most acute form, and the question is immediately raised, "What should be the attitude of boards of management to the many applications which must inevitably come to hand from resident medical officers for relief from their engagements in order to accept service in one of the various branches of military work open to medical men?" The desire will probably be general to afford every facility to resident medical officers to volunteer for service, but this at once opens up the important question, How are the hospitals to be efficiently staffed? It must be taken for granted that there are no newly qualified men left to take resident posts, so that advertising vacancies is not likely to be productive. To make matters more difficult, many of the members of the honorary medical and surgical staffs of hospitals are mobilised for duty in connection with hospital ships, Royal Red Cross, base hospitals, or other forms of official service, and cannot certainly give more time, and may have to give less, to their voluntary hospitals.

It seems more than possible that the difficulty may have to be met by the introduction of such means as the appointment of supernumerary physicians and surgeons from practitioners resident in the area of the hospitals, although it must not be overlooked that many practitioners have probably undertaken extra work for colleagues who have already been called up for service. As the difficulty is one which will seriously affect hospitals generally, we should like to have the views of our readers upon this important problem, and their suggestions for meeting the temporary difficulties of an unusual situation.

